# A necrotic primary central nervous system lymphoma in immunocompetent patient with MYC and BCL6 rearrangements (double-hit lymphoma): a case report

**DOI:** 10.1093/omcr/omad026

**Published:** 2023-03-25

**Authors:** Nabil C N Khalil, Bashaer Imad Iwaiwi, Shurooq Hammad, Afnan W M Jobran, Saeed Itkaidek, Elias Edward Lahham

**Affiliations:** Research Department, Faculty of Medicine, Al-Quds University, Jerusalem, Palestine; Research Department, Faculty of Medicine, Al-Quds University, Jerusalem, Palestine; Research Department, Faculty of Medicine, Al-Quds University, Jerusalem, Palestine; Research Department, Faculty of Medicine, Al-Quds University, Jerusalem, Palestine; Department of Neurosurgery, Al-Ahli Hospital, Hebron, Palestine; Research Department, Faculty of Medicine, Al-Quds University, Jerusalem, Palestine

## Abstract

Primary Central Nervous System Lymphoma (PCNSL) is a rare, aggressive extranodal non-Hodgkin lymphoma. It is critical to get a diagnosis and start therapy as soon as possible to improve clinical results. Despite a new medicinal strategy that has increased survivability, the survival rate is still quite low. This report presents a new case of PCNSL that appears in an immunocompetent patient with two different rare genetic rearrangements and a necrotic histological appearance.

## INTRODUCTION

Primary Central Nervous System Lymphoma (PCNSL) is a rare highly aggressive extra nodal non-Hodgkin lymphoma that affects the brain and spinal cord without systemic involvement. Most PCNSLs are diffuse large B-cell lymphomas (DLBCLs), accounts for 2–4% of CNS tumors and 4–6% of all extranodal lymphomas. Currently, immunocompetent patients represent the vast majority of patients with PCNSL. In addition, these patients are often diagnosed, mostly between the ages of 50 and 70 years [1]. This case study will present a 54-year-old woman diagnosed with high-grade B-cell lymphoma with *MYC* and *BCL6* rearrangements (double-hit lymphoma) rearrangements and a necrotic histological appearance. These results add a new case of rare MYC/BCL6 double-hit PCNSL to the literature.

## CASE PRESENTATION

**Figure 1:**
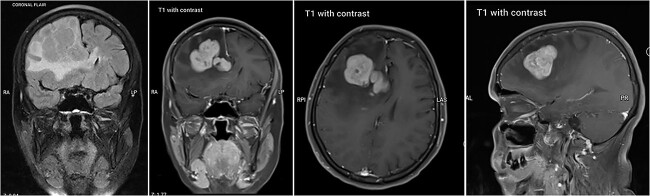
Brain MRI showing right-sided fronto-parietal mass.

A 54-year-old Middle Eastern female patient presented to the emergency room for the evaluation of recurrent episodes of falling down for the past 6 months with preserved consciousness level. She had intermittent episodes of diffuse, progressive headaches lasting for 30 min. Her past medical history was unremarkable. Neurological examination revealed mildly decrease in Power (4/5), hyperreflexia and a positive Babinski sign on the left side, with left side facial palsy. A brain CT scan performed at the local hospital showed a space-occupying lesion. The patient was then transferred to our hospital. After admission, a brain MRI was done and showed a large intra-axial lobulated solid mass centered in the right fronto-parietal region, measuring about 4^*^5.5^*^3.5 cm. The mass looked heterogeneous and hypo-intense on T1, hyper-intense on T2. The mass abuts the body and rostrum of the corpus callosum. There was vasogenic edema in the right front-parietal region, which extends into the contra-lateral side via the rostrum of the corpus callosum showing multiple hyper-intense FLAIR signals in the left periventricular region. There was compression of the anterior horn of the right lateral ventricle with midline shifting to the left side by 8 mm with subfalcine herniation. The mass looked highly vascular with no calcification or bleeding. CSF analysis was not done due to a mass occupying lesion with midline shifting on imaging (Fig. 1).

**Figure 2:**
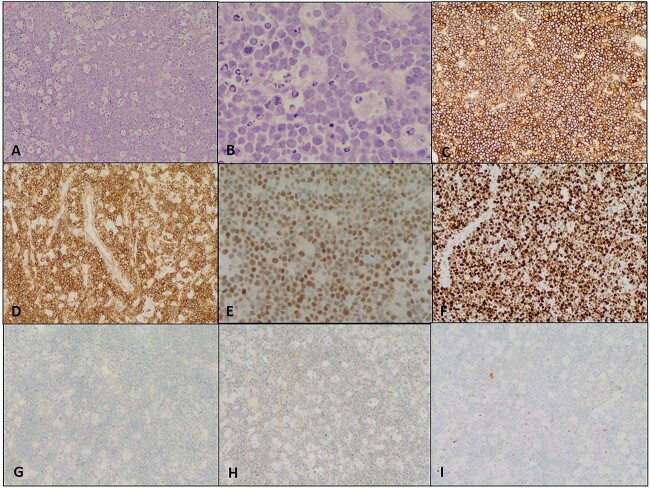
High-grade B-cell lymphoma. (**A**) Cellular tumor with monotonous lymphoid cells and starry sky appearance (H&E, 10×). (**B**) Atypical lymphoid cells are round to oval, medium-sized with minimal cytoplasm and fine nuclear chromatin with occasional small nucleoli. Mitotic activity and karyorrhectic cellular debris are increased (H&E, 40×); by immunohistochemistry, the lymphoma cells are positive for (**C**) CD20 (10×), (**D**) BCL2 (10×) and (**E**) BCL6 (20×). (**F**) Ki67 proliferation index in 98–100%. The lymphoma cells are negative for (**G**) CD10 (10×), (**H**) TdT (10×) and (**I**) Cyclin D1 (10×). Paraffin sections were used for paraffin-embedded tissue section—fluorescent in situ hybridization, revealed a rearrangement Translocation involving MYC and BCL6 was identified of the MYC/(8q24.2) split-signal positive rate was 93.5%, a rearrangement of the BCL6/(3q27.3) gene in 97.7% and a gain of BCL2/(18q21.3) in 69.5% of nuclei. There was no rearrangement of the BCL2 gene. According to these results, the diagnosis of PCNSL was made after a review in a multidisciplinary setting by a team involving in addition of neurosurgeon, a hematologist, a radiologist, a pathologist, etc.

**Figure 3:**
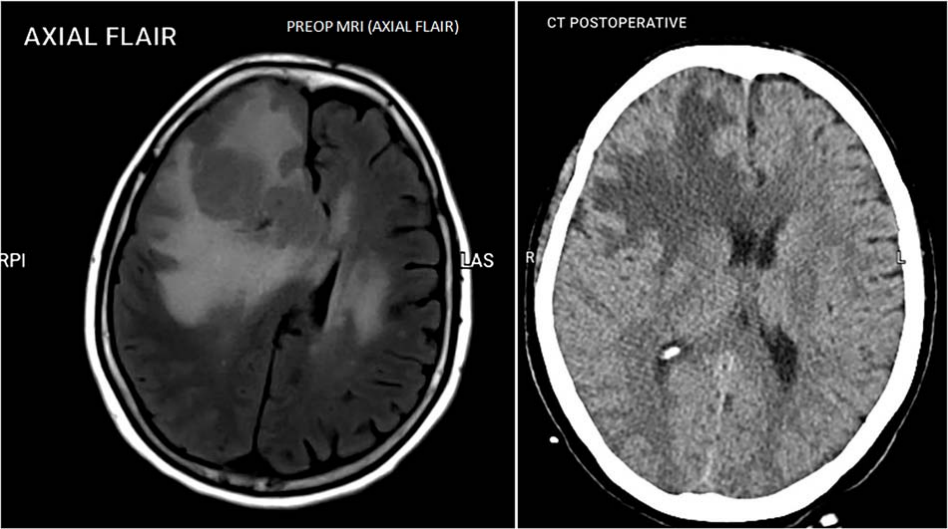
Pre-op vs. post-op brain CT scan.

A resection and biopsy were done. The lesion was firm with mild vascularity, highly necrotic and poorly defined with no clear-cut demarcation from the surrounding structures. Corticosteroid was given after a stereotactic brain biopsy. The patient clinically improved, she managed to walk independently, became free of headaches and her facial palsy improved*.* Microscopic features and immune-histochemical profile showed: positive for BCL2 and negative for CD10 raising the possibility for high-grade B-cell lymphoma (HGBCL), although there were frequent tangible body macrophages resulting in a starry sky appearance which is typically seen in Burkitt lymphoma. CD20, MYC, BCL-2 and BCL-6 were positive, while CD3, CD10, TdT and cyclin D1 immunostains were negative. Ki67 proliferation index is 98–100%. These all go along with HGBCL (Figs 2 and 3).

The International Extranodal Lymphoma Study Group Score (IELSG) for the patient was 3/5, classified as a moderate-risk patient, correlating with 2-year survival rates of 48% [2]. After the diagnosis was confirmed, a total of 6 cycles of chemotherapy were given, IV rituximab (375 mg/m^2^ 600 mg d1) and IV high-dose methotrexate (HD-MTX) (3 mg/m^2^ 4.89 g d2), with supportive treatment. We did not proceed with MATRix and ASCT due to financial, resource and political issues, in the context of the COVID-19 pandemic. The patient showed dramatic improvement in her symptoms of limb weakness, could take care of herself and could walk with no assistance. Cerebrospinal fluid cytology was negative. Due to financial concerns, a PET CT and bone marrow were not performed. A whole-body CT scan was done and she was diagnosed with PCNSL. The follow-up brain CT scan showed a decrease in midline shifting and frontoparietal vasogenic edema.

## DISCUSSION/CONCLUSION

PCNSL is a rare extranodal non-Hodgkin lymphoma [2]. The gold standard for diagnosis is histopathological evaluation [3]. Genetic rearrangement in PCNSL carries a prognostic relevance and possible therapeutic potential. The majority of double-hit lymphomas (75%) are marked by MYC plus BCL2 translocations, while BCL6 translocation is less common. Lymphomas that morphologically resemble DLBCL but contain translocation in the MYC gene with rearrangement in BCL2 or BCL6 are described as double-hit lymphomas and considered high-grade B cell lymphomas rather than DLBCL [4, 5]. Approximately 5% of these lymphomas are double-hit lymphomas which are considered very aggressive tumors with worse prognosis if treated as DLBCL and are associated with significantly poorer progression-free survival (PFS) and earlier relapse [4]. Without treatment, the survival is several weeks to months. However, treated patients have a 5-year survival of 30% [ 4]. A case series suggests that the therapy of these lymphomas needs to be targeted against the MYC gene and its proteins, as raising the dose of conventional chemotherapy has no effect on overall survival (OS) [5]. The IELSG score is used to estimate survival using five parameters (age, lactate dehydrogenase level, CSF protein concentration, Eastern Cooperative Oncology Group performance score and deep brain involvement). Low, moderate and high-risk patients have 80, 48 and 15% 2-year survival, respectively [2, 6]. Historically, whole-brain radiotherapy (WBRT) was used in the treatment of PCNSL. Despite the high response rate, it has limited survival benefits ranging between 12 and 18 months. Moreover, the neurotoxic effect that WBRT may have on patients has limited its use [2]. Different chemotherapies were ineffective as they do not pass the brain–blood-barrier. Recently, the 7-year results of the IELSG32 trial were analyzed, providing the dramatic effect of the MATRix regimen (methotrexate, cytarabine, thiotepa and rituximab) with overall survival (OS) of 56%, MATRix plus consolidation therapy was associated with great durable outcomes and did a paradigm shift in PCNSL treatment [7]. MATRix has a better PFS and OS (52 and 56%) compared with the combination of HD-MTX/HD-AraC (20 and 26%) or rituximab/HD-MTX/HD-AraC (29 and 37%), respectively [7]. PCNSL was treated with WBRT alone; the OS was 16 months, however, improved to 30 months using HD-MTX before WBRT [8]. More recently, the (IELSG43) trial demonstrated that high-dose chemotherapy followed by autologous hematopoietic stem cell transplant (HDT/ASCT) significantly improved outcomes compared with non-myeloablative chemoimmunotherapy [9]. Patients undergoing surgical resection of a single lesion had a higher chance of complete remission 6 months postoperatively compared with biopsy. However, the OS and complication incidence were not statistically significant [10].

## Data Availability

The data used to support the findings of this study are included within the article.
