# Correction to: A necrotic primary central nervous system lymphoma in immunocompetent patient with MYC and BCL6 rearrangements (double-hit lymphoma): a case report

**DOI:** 10.1093/omcr/omad069

**Published:** 2023-06-26

This is a correction to: Nabil C N Khalil and others, A necrotic primary central nervous system lymphoma in immunocompetent patient with MYC and BCL6 rearrangements (double-hit lymphoma): a case report, *Oxford Medical Case Reports*, Volume 2023, Issue 3, March 2023, omad026, https://doi.org/10.1093/omcr/omad026

The following changes have been made to the originally published article. An acknowledgements section has been added to read: ``ACKNOWLEDGEMENTS The authors wish to thank Dr Sami Bannoura at Al-Ahli Hospital for providing the MRI and CT images presented herein.''

This lacuna was emended in the article.

